# Impact of a Ketogenic Diet on Sporadic Inclusion Body Myositis: A Case Study

**DOI:** 10.3389/fneur.2020.582402

**Published:** 2020-11-05

**Authors:** Matthew C. L. Phillips, Deborah K. J. Murtagh, Fouzia Ziad, Samuel E. Johnston, Ben G. Moon

**Affiliations:** ^1^Department of Neurology, Waikato Hospital, Hamilton, New Zealand; ^2^Healthy Kitchen Christchurch Ltd., Hamilton, New Zealand; ^3^Department of Pathology, Waikato Hospital, Hamilton, New Zealand; ^4^Department of Older Persons and Rehabilitation, Waikato Hospital, Hamilton, New Zealand; ^5^Department of Radiology, Waikato Hospital, Hamilton, New Zealand

**Keywords:** inclusion body myositis, ketogenic diet, inflammation, degeneration, muscle

## Abstract

Sporadic inclusion body myositis (IBM) is a chronic inflammatory and degenerative muscle disease with limited treatment options; no therapy can alter its natural course. Ketogenic diets are theoretically capable of suppressing inflammation, enhancing cell bioenergetics, alleviating mitochondria dysfunction, and stimulating autophagy, which may be beneficial in IBM. We report the case of a 52-year-old woman with worsening IBM who pursued a modified ketogenic diet for 1 year. Adverse effects were mild and resolved 3 weeks into the diet. Prior to starting her ketogenic diet, despite the use of a walking stick at all times, she was experiencing one to two falls per week as well as swallowing difficulties, musculoskeletal pain, and depression. Moreover, magnetic resonance imaging (MRI) of the bilateral thighs during the year prior to the diet indicated worsening muscle inflammation and a 14% decrease in thigh muscle volume, which corresponded to a 4% decrease in the ratio of thigh muscle to thigh total volume. After 1 year on her ketogenic diet, our patient regained independent walking, and her swallowing difficulties, pain, and depression resolved. She maintained her strength, improved in every test of function, enhanced her quality of life, and lowered her blood creatine kinase. MRI of the bilateral thighs during the year of the diet indicated stabilized muscle inflammation and a 2.9% decrease in thigh muscle volume, which in the context of diet-induced fat loss corresponded to a sustained 1% increase in the ratio of thigh muscle to thigh total volume. This case is unique in that a ketogenic diet was utilized as the primary treatment strategy for a patient with confirmed IBM, culminating in substantial clinical improvement, stabilized muscle inflammation, and a slowed rate of muscle atrophy. Our patient has remained on her ketogenic diet for over 2 years now and continues to enjoy a full and independent life.

## Introduction

Sporadic inclusion body myositis (IBM) is the most common acquired myopathy in people over 50 years of age (1). Pathologically, IBM manifests as a unique triad consisting of an inflammatory infiltrate, a degenerative component, and mitochondria anomalies (1–3). The inflammatory infiltrate consists of CD8^+^ T cells that surround and invade muscle fibers expressing major histocompatibility complex class I (MHC-1). There is also a degenerative component, which is comprised of cytoplasmic and intranuclear inclusions containing aggregates of various types of proteins, as well as numerous mitochondria anomalies, as demonstrated by an abnormal increase in the number of cytochrome oxidase-negative muscle fibers. It has been hypothesized that many of these pathological features stem from impaired regulation of autophagy, a cellular mechanism responsible for the selective degradation of long-lived proteins and aged, dysfunctional mitochondria (4). Clinically, patients with IBM typically experience progressive, asymmetric wasting and weakness affecting the distal upper and proximal lower limbs, and frequently dysphagia (2, 3). Strength declines by about 5% per year (5), with lower limb muscles most severely affected; quadriceps strength may decline by as much as 27.9% per year (6). Functional ability declines by 13.8% per year, with a predisposition to falls being one of the most disabling features such that 7 years after disease onset, 63% of patients have lost independent walking (6). IBM does not respond to immunotherapy and exercise programs show mixed results (1–3). Novel therapeutic strategies are needed for IBM.

Ketogenic diets are high-fat, adequate-protein, and low-carbohydrate diets that stimulate the body to mimic a fasted metabolic state (7). After several days on such a diet, the human body enters a state of physiological ketosis characterized by low-blood glucose levels, emptied liver glycogen stores, and hepatic production of ketones that serve as a major energy source for brain and muscle. Ketogenic diets are theoretically capable of mitigating the pathology of IBM by suppressing inflammation, enhancing cell bioenergetics, alleviating mitochondria anomalies, and stimulating autophagy (7, 8). To our knowledge, a ketogenic diet has never been implemented as a therapeutic strategy in IBM.

## Case Study

**Figure 1:**
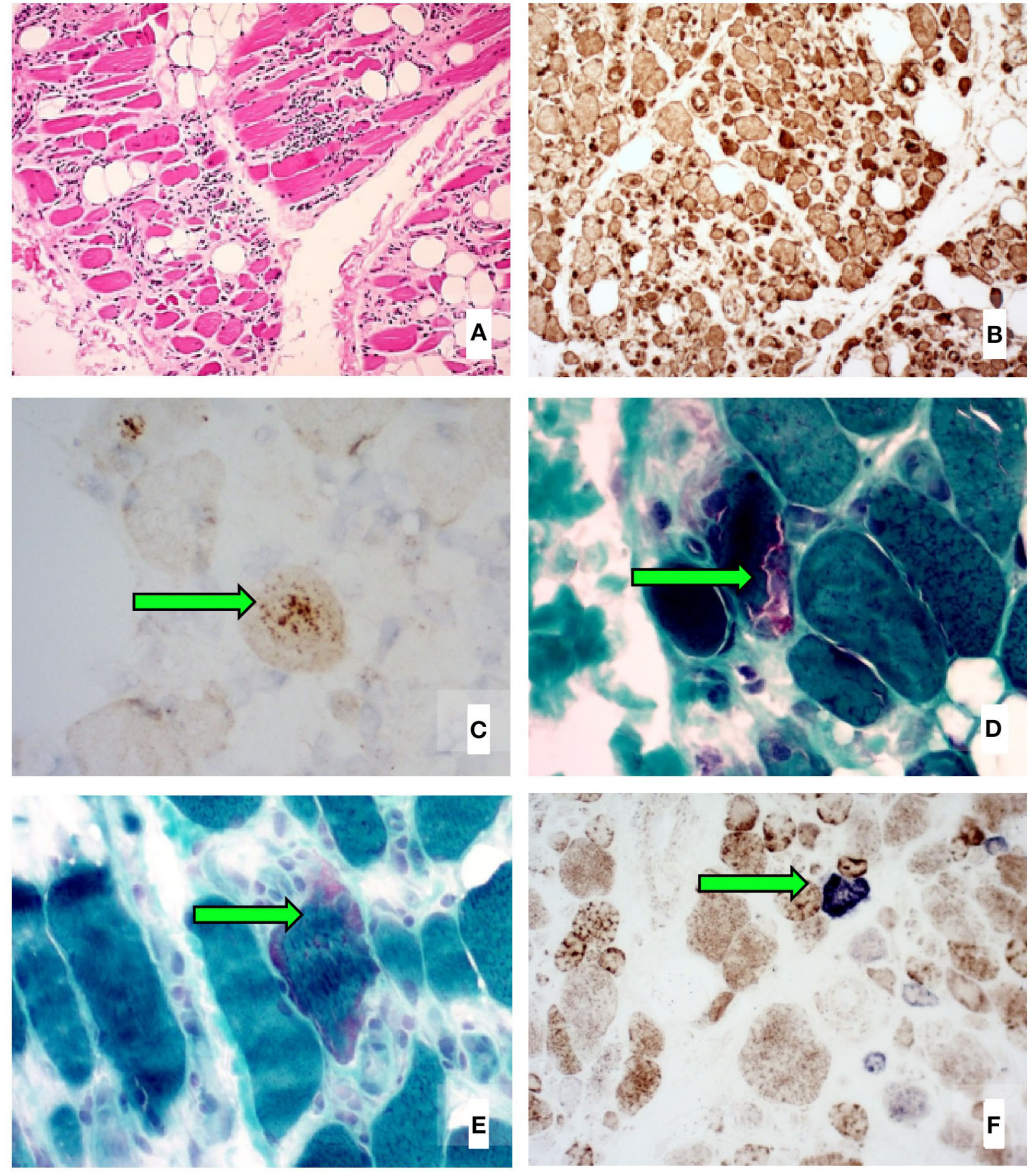
Left vastus lateralis biopsy histological images showing **(A)** marked variability in muscle fiber size, increased interstitial connective tissue, and several inflammatory infiltrates (H&E stain), **(B)** widespread sarcolemmal upregulation of MHC-1 (immunohistochemical stain), **(C)** p62-positive aggregates in vacuolated muscle fibers (immunohistochemical stain), **(D)** rimmed vacuoles (Gomori trichrome stain), **(E)** increased ragged red fibers (Gomori trichrome stain), and **(F)** increased numbers of cytochrome oxidase-negative muscle fibers for age (COX-SDH double stain).

We report the case of a 52-year-old female schoolteacher who initially presented with 2 years of progressive, asymmetric weakness affecting her distal upper and proximal lower limbs, resulting in an average of one fall per month. She also had difficulty swallowing food, leading to frequent choking episodes, as well as constant lower limb musculoskeletal pain. There was no other significant medical history and no family history of neuromuscular disease. She required daily tramadol and paracetamol for her pain. She weighed 66 kg. A neurological examination revealed weakness in shoulder abduction (4/5 bilaterally), elbow flexion and extension (4+/5 bilaterally), wrist flexion (4/5 bilaterally), wrist extension (4+/5 bilaterally), grip (4+/5 right, 4/5 left), hip flexion and extension (4+/5 bilaterally), knee flexion (4/5 bilaterally), and knee extension (4/5 right, 3+/5 left). Electromyography of the lower limbs demonstrated infrequent fibrillation potentials and a chronic mixed pattern (consisting of both short- and long-duration motor unit action potentials) affecting the iliopsoas, vastus lateralis, and tibialis anterior muscles. Serial blood creatine kinase (CK) levels were elevated at 1,000–1,500 U/L (normal range, 30–180 U/L). Biopsy of the left vastus lateralis muscle revealed a marked variability in muscle fiber size, increased interstitial connective tissue, an endomysial and perivascular infiltrate composed of lymphocytes and histiocytes invading necrotic and non-necrotic fibers, widespread upregulation of MHC-1, p62-positive inclusions, rimmed vacuoles, increased ragged red fibers, and increased numbers of cytochrome oxidase-negative muscle fibers for age (Figure 1). Magnetic resonance imaging (MRI) of the bilateral thighs revealed significant short-T1 inversion recovery (STIR) signal changes and loss of muscle volume affecting the pelvic and thigh muscles (with the quadriceps muscles most severely affected, more so on the left) consistent with myositis and muscle atrophy. Our patient was subsequently diagnosed with clinicopathologically defined IBM based on the 2011 European Neuromuscular Centre diagnostic criteria (9).

Despite commencing a regular, 3-day-per-week resistance exercise program, our patient had clearly declined by the time of her 1-year review. By this point, she required a walking stick at all times; despite the walking stick, she was falling 1–2 times per week. Her swallowing difficulties and choking episodes were ongoing, as was her musculoskeletal pain. She expressed concern regarding the uncertainty of her future and was clinically depressed. MRI of the bilateral thighs revealed increased STIR signal changes (with several new muscles affected), a 14% decrease in thigh muscle volume, and a 4% loss in the ratio of thigh muscle to thigh total volume compared to the diagnosis scan, consistent with ongoing myositis and muscle atrophy. Given the profound deterioration and lack of treatment options, a ketogenic diet intervention was offered; after all foreseeable risks and benefits had been explained, she chose this course.

**Figure 2:**
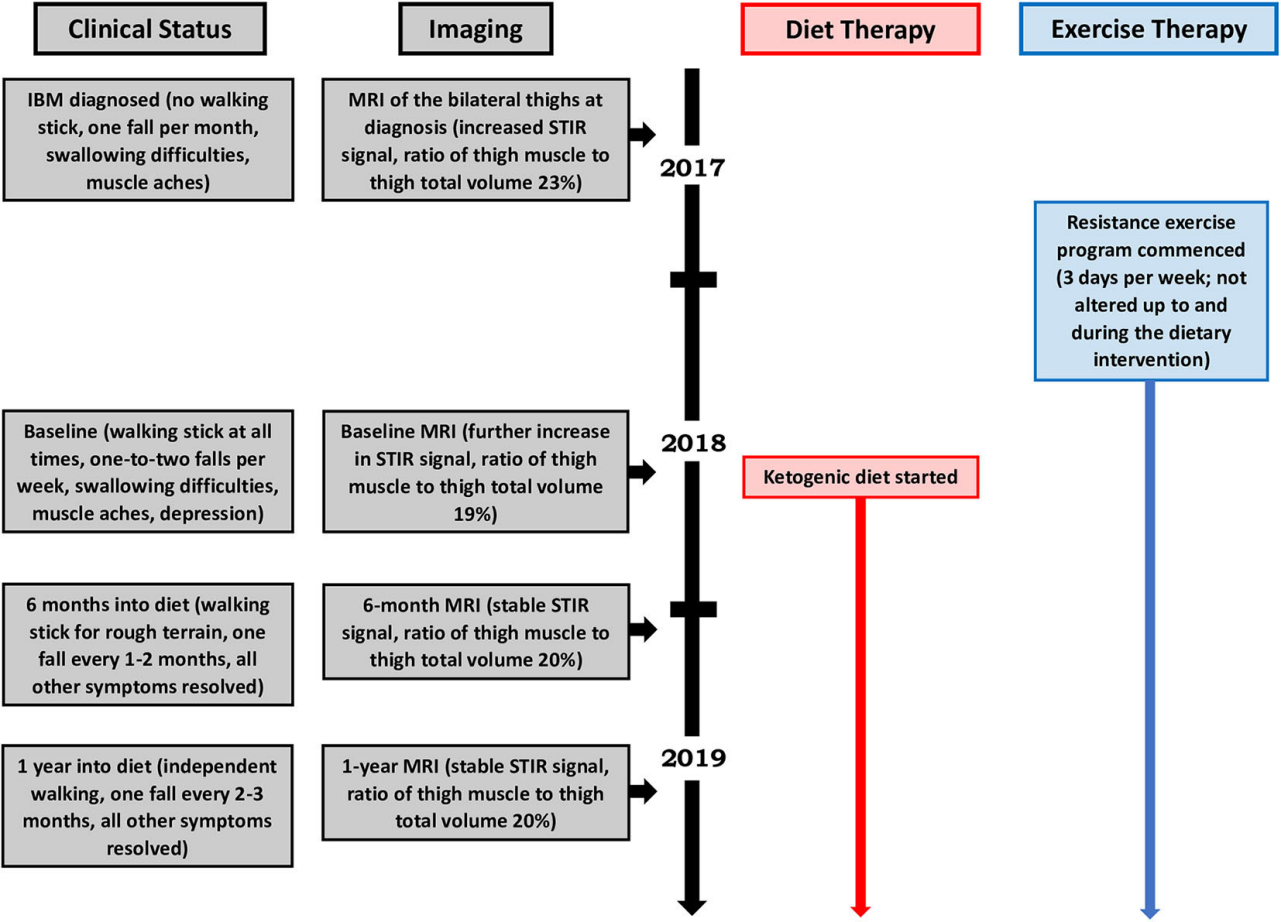
Patient timeline.

Our patient's health and neurological status were regularly monitored by a neurologist during the diet intervention, which consisted of a modified ketogenic diet (60% fat, 30% protein, 5% fiber, and 5% net carbohydrate by weight, comprised of green vegetables, meats, eggs, nuts, seeds, creams, and natural oils) (Figure 2). No further medications were administered and her exercise program remained unaltered throughout the intervention. She monitored and recorded her blood glucose and beta-hydroxybutyrate (BHB) levels (Freestyle Neo; Abbott Diabetes Care, Whitney, UK) three times per week (10). All adverse effects were documented.

**Figure 3:**
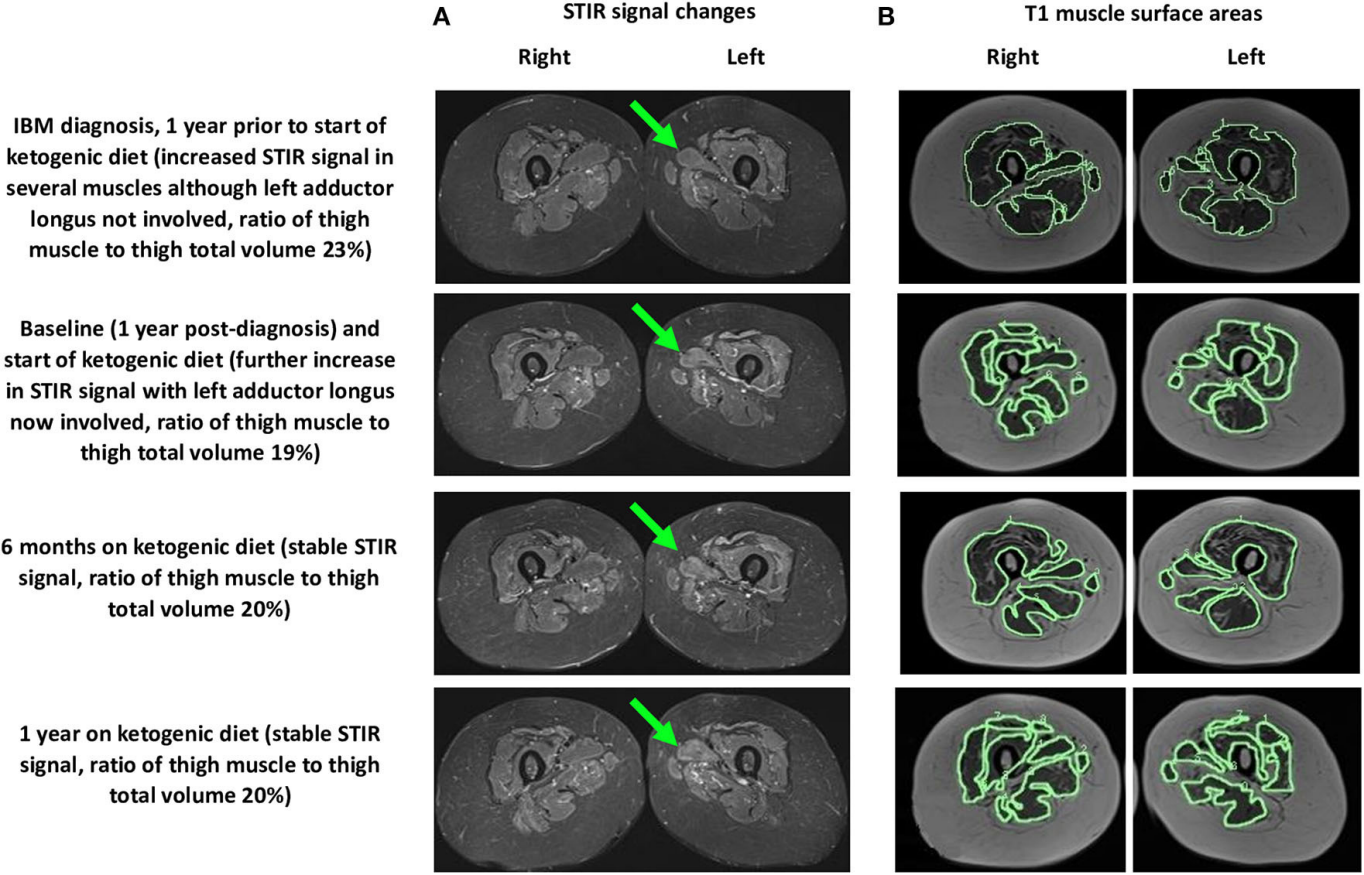
MRI (axial views) of the bilateral thighs showing thigh muscle **(A)** STIR signal changes (left adductor longus shown by green arrow) and **(B)** T1 muscle surface areas (outlined in green, only axial plane 3 of 5 is shown) at 1 year prior to starting the ketogenic diet and at baseline, 6 months, and 1 year after starting the diet.

We performed assessments at 6 months and 1 year after our patient started her modified ketogenic diet, which included measurements of our patient's clinical status, strength, function, quality of life, and blood markers, as well as radiological data on the degree of thigh muscle inflammation and volume loss (Table 1). To always maintain a consistent time interval between the most recent exercise workout and the day of assessment, she refrained from resistance exercise for exactly 1 week prior to each assessment, which was always administered on the same hour of the day in the same clothing and footwear. A treatment-blinded physiotherapist measured strength and function. Maximal strength was defined as the maximum weight displaced for one complete repetition in each of the chest press, pulldown, leg press, and bilateral upper limb grip testing on a multigym station (M5 Multi Gym, Inspire Fitness, Corona, CA) (11). Function was measured using the get-up-and-go, 6-min walk, and stair-climb tests (for each measure, the average of two tests was taken) (12–14). Quality of life was measured using the Functional Assessment of Chronic Illness Therapy-Fatigue (FACIT-F) subscale (scores range from 0 to 52, with higher numbers indicating better quality of life) (15). Blood CK and other metabolic markers were recorded. A treatment-blinded radiologist analyzed serial MRI scans of the bilateral thighs so as to assess the degree of muscle inflammation and volume loss (Figure 3). Thigh muscle inflammation was measured using a qualitative analysis of STIR signal changes. Thigh muscle volume was measured using volume-estimation analysis of T1-weighted images, in which each scan was divided into five axial planes along a vertical axis (with each plane separated by exactly 5 cm), after which the same treatment-blinded radiologist manually measured thigh muscle surface area and thigh total surface area within each plane; the cross-sectional surface areas were then multiplied by the distance between each plane to provide estimates of thigh muscle volume, thigh total volume, and the ratio of thigh muscle to thigh total volume.

**Table 1:** Clinical status, strength, function, quality of life, blood markers, and MRI assessment data at baseline, 6 months, and 1 year after starting the ketogenic diet.

	**Baseline (1 year post-diagnosis, start of the diet)**	**6 months into the diet**	**1 year into the diet**
**Clinical status**			
Need for a walking stick	Needed at all times	Needed for rough terrain	Independent walking
Frequency of falls	One to two falls per week	One fall every 1–2 months	One fall every 2–3 months
Swallowing difficulties and choking	Ongoing	Resolved	Resolved
Muscle aches	Ongoing	Resolved	Resolved
Mood	Clinical depression	Resolved	Resolved
**Strength**			
Chest press (kg)	14	9	14
Pulldown (kg)	14	18	23
Leg press (kg)	18	18	18
Grip test, right (kg)	22	20	20
Grip test, left (kg)	14	13	13
**Function**			
Get-up-and-go (s)	15.6	11.6	11.1
6-min walk (m)	327.5	368.0	373.0
Stair climb (s)	36.4	30.7	28.6
**Quality of life**			
FACIT-F subscale	40	48	48
**Blood markers**			
Hemoglobin (mmol/L)	124	120	124
Creatinine (mmol/L)	58	49	35
CK (mmol/L)	1,370	958	791
HbA1C (mmol/mol)	37	33	32
Triglycerides (mmol/L)	1.6	1.8	1.3
HDL (mmol/L)	1.44	1.75	1.71
LDL (mmol/L)	2.5	2.1	2.4
Cholesterol (mmol/L)	4.7	4.7	4.7
CRP (mmol/L)	1.1	1.9	1.3
**MRI**			
Short-T1 inversion recovery (STIR) signal changes (signal intensity and whether or not new muscles were affected)	Increased STIR signal since diagnosis 1 year previously, new muscles affected	Stable STIR signal, no new muscles affected	Stable STIR signal, no new muscles affected
Thigh muscle volume (cm^3^)	2,252 (2,627 at diagnosis)	2,295	2,184
Thigh total volume (cm^3^)	11,861 (11,411 at diagnosis)	11,295	10,962
Ratio of thigh muscle to thigh total volume	19% (23% at diagnosis)	20%	20%

During the year of her modified ketogenic diet, our patient improved. After only 2 weeks on the diet, her musculoskeletal pain disappeared and she ceased all tramadol and paracetamol. By the end of week 4, her anxiety and depressive symptoms had resolved. By the end of 1 year, she had regained independent walking and no longer required a walking stick. The frequency of her falls had decreased to one fall every 2–3 months. She no longer experienced swallowing difficulties or choking episodes. Quantitative testing revealed generally maintained muscle strength and improvements in all three tests of function, namely the get-up-and-go (which decreased from 15.6 to 11.1 s by the end of 1 year, representing a 29% improvement from baseline), 6-min walk (which increased from 327.5 to 373.0 m, representing a 14% improvement), and stair climb (which decreased from 36.4 to 28.6 s, representing a 22% improvement). She also demonstrated an enhanced quality of life on the FACIT-F subscale (which increased from 40 to 48 points by the end of 1 year, representing a 20% improvement from baseline) as well as a lowered blood CK (from 1,370 to 791 mmol/L, representing a 42% improvement). MRI of the bilateral thighs showed stable STIR signal changes (with no new muscles affected), a 2.9% decrease in thigh muscle volume, and a sustained 1% gain in the ratio of thigh muscle to thigh total volume compared to the baseline scan.

After 1 year on a modified ketogenic diet, our patient weighed 59 kg and her mean blood glucose and BHB levels (±standard deviation) were measured at 5.10 ± 0.59 and 2.28 ± 0.77 mmol/L, respectively. The only adverse effects she experienced were fatigue and a mild headache, both of which resolved after 3 weeks on the diet.

## Discussion

This case is unique in that a patient with confirmed, disabling IBM was successfully managed with a modified ketogenic diet, culminating in independent walking and the complete resolution of her swallowing difficulties, pain, and depression. Our patient maintained her strength, improved in every test of function, enhanced her quality of life, and progressively lowered her blood CK. Her thigh muscle inflammation stabilized. During the year prior to starting her ketogenic diet, she lost 14% of her thigh muscle volume, corresponding to a decrease of 4% in her ratio of thigh muscle to thigh total volume; in contrast, during the year of the diet, despite losing 11% of her body weight, she lost only 2.9% of her thigh muscle volume, corresponding to a sustained 1% increase in her ratio of thigh muscle to thigh total volume. Adverse effects were mild and resolved 3 weeks into the diet. Our patient has now remained on her ketogenic diet for over 2 years and continues to derive benefit from it. She enjoys a full and independent life and requires no medications.

Ketogenic diets are theoretically capable of mitigating the pathology of IBM by suppressing inflammation, enhancing cell bioenergetics, alleviating mitochondria anomalies, and stimulating autophagy. Several mechanisms exist by which ketogenic diets might suppress inflammation (8). Compared to glucose metabolism, ketone metabolism generates fewer reactive oxygen species (8). Moreover, the primary blood ketone, BHB, suppresses the activation of the NLRP3 inflammasome, an important inflammatory mediator (16), and acts as an endogenous inhibitor of class 1 histone deacetylases, thereby increasing transcription rates for genes encoding oxidative stress resistance factors (17). Furthermore, ketogenic diets increase the production of the anti-inflammatory neuromodulator adenosine (8). These anti-inflammatory mechanisms are supported by findings in animal models—for example, ketones are protective against oxidative injury in rat neocortical neurons exposed to hydrogen peroxide or diamide (18), and peripheral inflammation is significantly reduced in rats fed a ketogenic diet (19). Beyond inflammation, ketogenic diets can potentially curtail degeneration by enhancing muscle cell bioenergetics (7). Compared to glucose, BHB is a more efficient energy source per unit oxygen due to an increased free energy of adenosine triphosphate (ATP) hydrolysis (20). BHB also increases cell ATP production by replenishing citric acid cycle intermediates—for example, compared to controls, mice on a 3-week ketogenic diet display higher concentrations of citrate and α-ketoglutarate, as well as a higher ATP/ADP ratio (21). With regard to mitochondria anomalies, a ketogenic diet decreases the amount of cytochrome oxidase-negative muscle fibers in a mouse model of mitochondrial myopathy, thus slowing the progression of the myopathy (22). Lastly, fasting-induced autophagy is well-described in animal models (23); since fasting and ketogenic diets activate similar pathways, the latter may also stimulate autophagy, potentially lessening the accumulation of autophagy-associated proteins.

Our patient's serial MRI findings are supportive of the possibility that her modified ketogenic diet exerted anti-inflammatory and anti-degenerative effects on her IBM. During the year prior to starting her ketogenic diet, the STIR signal changes increased in her thigh muscles, with several new muscles affected; in contrast, during the year of her ketogenic diet, the STIR signal changes remained stable, with no new muscles affected. These findings suggest that the diet prevented inflammation from worsening in affected muscles while also preventing inflammation from emerging in unaffected muscles. Moreover, during the year prior to starting her ketogenic diet, our patient lost 14% of her thigh muscle volume (thigh muscle volume decreased from 2,627 to 2,252 cm^3^, representing a loss of 375 cm^3^) and the ratio of her thigh muscle to thigh total volume decreased by 4%; in contrast, during the year of her ketogenic diet, despite losing 11% of her body weight, she lost only 2.9% of her thigh muscle volume (2,252 to 2,184 cm^3^, representing a loss of 68 cm^3^), which translates to only 18% of the thigh muscle lost in the pre-diet year. Given that the 2.9% thigh muscle volume loss occurred in the context of an 11% decrease in body weight (most of which would have been fat loss), our patient's ratio of thigh muscle to thigh total volume actually increased by 1% during the year of her ketogenic diet. Furthermore, the 2.9% decrease in thigh muscle volume occurred in the context of a marked 42% decrease in blood CK; the magnitude of the CK decrease is best explained by a substantially slowed rate of muscle degradation (rather than ongoing atrophy). Collectively, these findings are somewhat remarkable considering that our patient's thigh muscles were her most severely affected muscles.

Several factors may have contributed to our patient's improved swallowing, walking, and function. She sustained a moderately high level of ketosis, which presumably provided a diet-mediated bioenergetic advantage for her muscle cells and mitochondria, culminating in increased muscle energy efficiency and function. However, it is also likely that the 7 kg of weight loss our patient experienced during the year of her ketogenic diet lessened the weight burden on her lower limbs, which would have contributed to fewer falls and improved function; given that weight loss is a common side effect seen in ketogenic diets, this represents an indirect effect of the diet that probably had a positive impact on our patient (albeit not through any specific effect on muscle inflammation or degeneration). Another possibility is that our patient adapted her walking pattern to her weakness; adaptation has been described in some patients with IBM and may result in fewer falls (24). However, although weight loss and adaptation could have contributed to our patient's improved walking and performance on tests of function, neither weight loss nor adaptation can fully explain the resolution of our patient's swallowing difficulties, pain, and depression, nor can they explain her generally maintained strength, progressively lowered blood CK, stable STIR signal changes, and slowed rate of muscle loss.

Since this study involved one patient, it is difficult to precisely determine the mechanism of improvement—for example, although our patient's modified ketogenic diet may have improved her muscle function by conferring a bioenergetic advantage, it may have also done so through its resolution of other symptoms, such as her musculoskeletal pain or depression. Despite this uncertainty, the fact remains that the only change made in our patient's life over the course of 1 year was the ketogenic diet intervention; therefore, that intervention was—in some way—responsible for most or all of the improvements documented. An additional potential limitation of the approach used in this study is that conventional ketogenic diets have a reputation for being unpalatable, which is frequently their greatest impediment to long-term implementation. It is therefore important to note that we crafted a modified ketogenic diet based on our patient's cuisine preferences, which resulted in the creation of a highly palatable diet capable of sustaining a moderately high degree of physiological ketosis.

In conclusion, this case is unique in that a patient with confirmed, disabling IBM was successfully managed with a modified ketogenic diet, culminating in substantial clinical improvement, stabilized muscle inflammation, and a slowed rate of muscle atrophy. Our patient has continued to derive benefit from her ketogenic diet for over 2 years now, and enjoys a full and independent life. Given that patients with IBM are faced with limited treatment options, with no treatment capable of altering the natural course of this disabling disorder, the favorable outcome experienced by our patient holds promise. Further studies involving larger numbers of patients are indicated.

## Data Availability Statement

All datasets generated for this study are included in the article/supplementary material.

## Ethics Statement

Ethical review and approval was not required for the study on human participants in accordance with the local legislation and institutional requirements. The patients/participants provided their written informed consent to participate in this study. Written informed consent was obtained from the individual(s) for the publication of any potentially identifiable images or data included in this article.

## Author Contributions

MP: conception, design, interpretation, and write-up of final article. DM: diet implementation and advice and proof-reading of final article. FZ: histology analysis and advice and proof-reading of final article. SJ: strength and function analysis and advice and proof-reading of final article. BM: imaging analysis and advice and proof-reading of final article. All authors contributed to the article and approved the submitted version.

## Conflict of Interest

DM was employed by the company Healthy Kitchen Christchurch Ltd. The remaining authors declare that the research was conducted in the absence of any commercial or financial relationships that could be construed as a potential conflict of interest.
